# A new technique for uterine-preserving pelvic organ prolapse surgery: Laparoscopic rectus abdominis hysteropexy for uterine prolapse by comparing with traditional techniques

**DOI:** 10.1515/med-2023-0833

**Published:** 2023-10-25

**Authors:** Zijun Li, Yaqin Zheng, Fangrong Shen, Ming Liu, Ying Zhou

**Affiliations:** Department of Gynecology, The People’s Hospital of LongQuan, LongQuan, China; Clinical Laboratory Centre, The People’s Hospital of LongQuan, LongQuan, Zhejiang, China; Department of Gynecology, Soochow University Affiliated First Hospital, Suzhou, China; Department of Gynecology, Zhejiang Quhua Hospital, Quzhou, Zhejiang, China; Imagning Diagnosis Center, Zhejiang Quhua Hospital, Quzhou, Zhejiang, China

**Keywords:** pelvic organ prolapse, hysteropexy, hysterectomy

## Abstract

Contemporary understanding of the dynamic anatomy of pelvic floor support has led us to new conservative surgery for uterine prolapse (UP). In this study, we comprehensively evaluate the safety and feasibility of a new technique for uterine-preserving pelvic organ prolapse surgery: laparoscopic rectus abdominis hysteropexy for uterine prolapse (LRAHUP). A retrospective study was conducted between 2006 and 2016. Sixty-five women diagnosed with advanced prolapsed uterus were eligible and grouped into traditional vaginal surgery (TVS, *n* = 30) group and new laparoscopic surgery (NLS, *n* = 35) group. Evaluated items of 65 cases included surgery-related parameters and postoperative outcomes. Surgical safety evaluating indicators, including operation time, blood loss, postoperative hospitalized day, and operation complications, also showed great significant difference between two groups (*P* < 0.05). The subjective index of post-operative Pelvic Floor Distress Inventory-short form 20 scores and some objective anatomic outcomes all showed great difference between pre- and post-operation (*P* < 0.05). Although the TVL showed no difference between pre- and post-operation in the same group, the TVL displayed a remarkable elongation. And a remarkable tendency was a higher cumulative recurrence ratio in the TVS group and a shorter follow-up period in the NLS group. LRAHUP may be a good procedure to manage women with advanced prolapsed uterus.

## 1 Introduction

Traditionally, vaginal total hysterectomy (VTH) is the basic surgical approach for uterine prolapse (UP) during the past few decades. However, vaginal hysterectomy alone often fails to address the underlying deficiencies in pelvic support that cause new vault prolapse. In fact, the incidence of vaginal fornix prolapse in women with UP after simple hysterectomy and vaginal repair is as high as 40% [1,2]. In other words, hysterectomy may be both the reason for and outcome of vaginal fornix prolapse, especially for women patients with pelvic organ prolapse (POP); in addition, the uterus and cervix may have an important role in sexual function and well-being [3]. Contemporary understanding of the dynamic anatomy of pelvic floor support has led us to new conservative surgery for POP, especially for the management of UP [3,4]. The uterus itself does not play a key role in the pathogenesis of UP [2]. Therefore, simple hysterectomy should not be the prime treatment and may increase the recurrence ratio of vault prolapse [2]. Many different mesh-based uterine-sparing operative techniques have been created to treat UP and restore the anatomy of pelvic organs during the past few years, such as laparoscopic sacrohysteropexy/sacrocolpopexy, bilateral sacrospinous hysteropexy, or posterior intravaginal slingplasty with conservation of the uterus, which was the commonly performed procedure [5,6]. However, many mesh-related complications reported by Food and Drug Administration showed only a small part of iceberg of far more serious mesh-related complications [7,8]. So far, more and more gynecologists focused on this key problem, which is how to treat UP women with acceptance of no mesh and uterus-sparing POP surgery [9,10,11].

Therefore, in this study, we conducted a small case series to investigate the safety and feasibility in women patients with advanced POP by comparing with traditional vaginal surgery (TVS), who underwent the uterine-preserving POP surgery named as laparoscopic rectus abdominis hysteropexy for uterine prolapse (LRAHUP).

## 2 Materials and methods

### 2.1 Study design

This single-center, retrospective observational study was approved by the Ethics Committee of Zhe-Jiang Quhua Hospital (approved on January 01, 2015; approval no.ECZJQH-201500038), and the procedure has been registered as a new intervention and was approved by the New Procedures Clinical Governance Committee of Zhe-Jiang Quhua Hospital in Zhejiang Province (NPCGCZJQH-201500038). Prior to the study of this new technology, I had already conducted clinical trials on five patients. Although this new technology is simple and easy to learn, considering the level of proficiency and the missing part of the collected data, the five subjects were not included in this study. All clinical data of enrolled 65 case patients were completely collected, including pre-operative general characteristics including age, parity, body mass index, pre-operative Pelvic Floor Distress Inventory-short form 20 (PFDI-20) scores [12], POP quantification (POP-Q) stages (Ⅰ–Ⅳ) [13] and Old Laceration of Perineum (OLP) degree (I–IV) [14], some surgery-related evaluating indexes including operation time, blood loss, post-operative hospitalized day, operation complications, incidence of stress urinary incontinence (SUI) after operation, pre- and post-operative POP-Q anatomic parameters, post-operative PFDI-20 scores, and cumulative recurrence within the periods of follow-up time. Recurrence was defined as objective POP-Q stage ≥ stage II at the anterior/posterior/apical vaginal wall [13,15].

### 2.2 Enrolled patients

The subjects were patients with advanced UP who were scheduled to undergo traditional VTH or the uterine-preserving UP surgery (LRAHUP) in the Department of Gynecology at Zhe-Jiang Qu-Hua Hospital from January 2006 to January 2016. All patients who met the enrolled criteria were described as follows: diagnosed with grade Ⅲ or IV UP according to the POP-Q proposed by Bump et al. [13], postmenopausal women without uncontrollable respiratory disorders and intractable constipation, without significant uterine enlargement (e.g., uterine fibroids), without previous POP surgery, without concomitant medical problems (e.g., endometrial and cervical lesions), and complete information of patients (e.g., follow-up). Moreover, for the convenience of research, women patients with severe degree of SUI were not enrolled in this study, and only 65 case patients with light, moderate, and occult SUI were enrolled [16]. All enrolled patients gave a written informed consent before acceptance of this new technique. Finally, 65 case postmenopausal patients were enrolled and studied.

### 2.3 Procedure

Sixty-five case patients were divided into two groups according to acceptance of different surgical methods: 30 patients underwent traditional vaginal hysterectomy and anterior/posterior vaginal wall repair, which were categorized as the TVS group, and 35 patients underwent a new uterine-preserving POP surgery named as LRAHUP, except for anterior and posterior vaginal wall repair, which were categorized as new laparoscopic surgery (NLS) group. These two surgical procedures were prepared in the same way before the operation, and the operations were carried out by the same chief physician in our hospital. The detailed surgical strategies for different POP in different groups are displayed in Table 1. Furthermore, the specific surgical procedures were also concomitantly inspected, assessed, and determined by a deputy chief physician and the chief physician who performed the operation. Before and after the operation, the pelvic floor situation of all patients was evaluated by POP-Q. And a subjective index was assessed by PFDI-20 [12], which included Pelvic Organ Prolapse Distress Inventory-6 (POPDI-6), Colorectal Anal Distress Inventory-8 (CRADI-8), and Urinary Distress Inventory-6 (UDI-6). Moreover, some objectively assessed indexes were also collected, including anatomic parameters before and 1 week after operation, and different follow-up time. All 65 patients were completely under a 5-year follow-up after surgery for pelvic floor repaired-related complications and improvements of symptoms. All clinical data, including the general pre-operative characteristics and many operation-related evaluating indexes of 65 patients, were obtained from the women’s clinical and follow-up records.

**Table 1:** Summary of different surgical strategies for 65 – case POP women

Variables	TVS group			NLS group		
	II	III	IV	II	III	IV
AVWP^YES-SUI^	②+④	②+④+⑥	②+④+⑥	②+④	②+④+⑥	②+④+⑥
AVWP^NO-SUI^	②	②+⑥	②+⑥	②	②+⑥	②+⑥
UP	—	①+⑤	①+⑤	—	⑦	⑦
PVWP	③	③	③	③	③	③
OLP	⑧	⑧	⑧	⑧	⑧	⑧

AVWP^YES-SUI^ = anterior vaginal wall prolapse and with SUI, AVWP^NO-SUI^ = anterior vaginal wall prolapse without SUI, UP = uterine prolapse, PVWP = posterior vaginal wall prolapse, ① = VTH, ② = anterior vaginal wall repair, ③ = posterior vaginal wall repair, ④ = Kelly’s operation, ⑤ = suspension of uterosacral ligament, ⑥ = repair of bladder fascia, ⑦ = LARHUP, ⑧ = repair of perineum.

### 2.4 Key steps of these new procedures of LRAHUP

**Figure 1:**
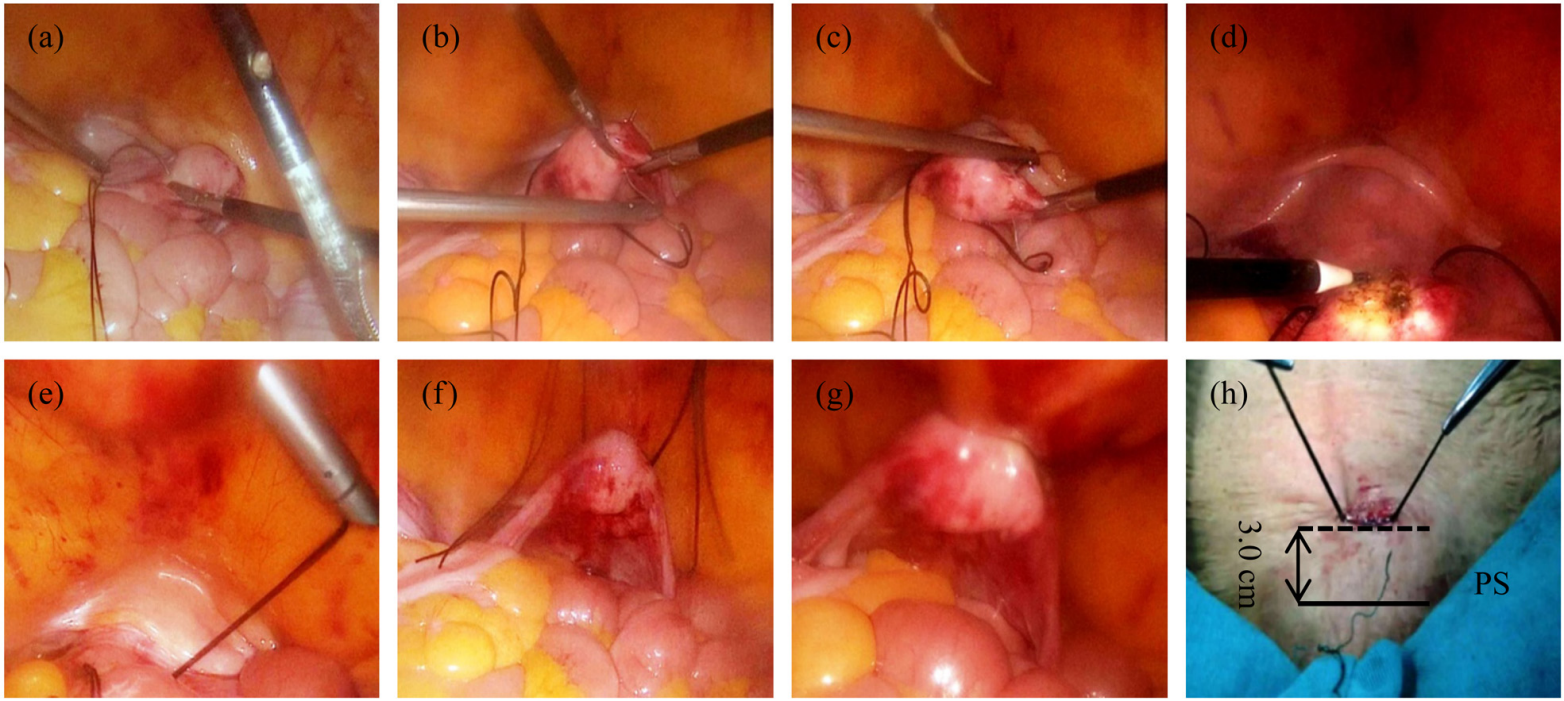
Illustrated surgical key steps for LRAHUP.

The details of the procedures of new technique for uterine-preserving POP surgery (LRAHUP) are described below:

1. First, to repair anterior and posterior vaginal wall repair as same as the TVS, repair of perineum and Kelly’s operation when necessary (e.g., light and moderate degree of SUI or occult SUI).
2. Laparoscopic suture of bilateral proximal uterine horn and uterine body joint: to suture with big round needle No.10 non-absorbable silk string, as shown in Figure 1a–c.
3. To make the adhesion surface of the anterior wall of uterine body burned and coagulated by monopole electro-coagulation (Figure 1d).
4. One-cut and two-tunnel location: to pull or push the bottom of the uterine body to evaluate and locate the cut and puncture position between 2.0 and 3.0 cm above the pubic symphysis (PS) (Figure 1h).
5. One-cut and two-tunnel puncture: first, one 15 mm diameter incision, second, a 5 mm diameter puncture instrument was first vertically punctured to the anterior sheath of rectus abdominis, and then it was punctured into the pelvic cavity on both sides with an inclined plane of 45° (Figure 1e and f).
6. To pull the suture out of pelvic cavity, tighten up and knot: a laparoscopic forceps passed through two tunnels separately and pull out both ends of the suture to the outside of the pelvic cavity and tighten the knot (Figure 1g and h).

### 2.5 Statistical analysis

Statistical analysis was carried out using SPSS 19.0 (SPSS Inc., Chicago, IL, USA). All measurement data, including pre-operative general characteristics, were analyzed using independent-samples *t*-test, and categorical counting data were analyzed using the chi-square or Pearson *x* ^2^ test and Fisher’s exact test. The constituent ratio was analyzed by Mann–Whitney *U*-test or Wilcoxon’s *W* test, and the comparisons of anatomic parameters were analyzed using analysis of variance (ANOVA). Moreover, construction and analysis of the interpolation line of relationship between the time of follow-up (lateral axis) and cumulative recurrence ratio (vertical axis) using graph tools of SPSS19.0; all *P*-values of <0.05 were considered statistically significant.


**Ethical approval:** All procedures performed in studies involving human participants were in accordance with the ethical standards of the institutional and/or national research committee and with the 1964 Helsinki Declaration and its later amendments or comparable ethical standards.

## 3 Results

### 3.1 General characteristics of 65 patients in different groups

The baseline characteristics of TVS and NLS groups were comparable, in terms of age, parity, body mass index, and PFDI-20 scores, including POPDI-6, CRADI-8, and UDI-6; there was no significant difference between two groups (all *P*-values >0.05) (Table 2).

**Table 2:** General characteristics of 65 case patients (*M* ± SD)

Variables	TVS group	NLS group	*t/*x* ^2^	*P*
Age (years)	65.83 ± 5.73	64.71 ± 5.14	*0.830	0.410
Parity	2.87 ± 0.97	3.34 ± 1.33	7.678	0.175
Body Mass Index (kg/cm^2^)	20.13 ± 1.52	20.38 ± 1.60	*−0.641	0.524
PFDI-20 (pre-operation scores)	93.50 ± 20.96	92.28 ± 20.09	46.222	0.169
POPDI-6	59.74 ± 7.20	57.01 ± 6.39	6.935	0.327
CRADI-8	8.17 ± 9.36	11.82 ± 8.02	9.231	0.100
UDI-6	25.57 ± 10.28	24.50 ± 12.87	12.317	0.550

^*^ *t* = independent-samples *T* test, *x* ^2^ = *x* square test. *M* ± SD = mean ± standard deviation.

### 3.2 Constituent ratio of POP-Q stage and different surgical strategies for POP

The constituent ratio of PU, PPVW, PAVW, and OLP showed separately no great significant difference between two groups (all *P*-value ＞0.05) (Table 3).

**Table 3:** Constituent ratio of different degrees of POP-Q, OLP, and SUI

Variables	TVS group	NLS group	*Z*	P*
PU			−0.520	0.603
III (*n* = 57)	27(27/30)	30(30/35)		
IV (*n* = 8)	3(3/30)	5(5/35)		
PPVW			−0.433	0.665
II (*n* = 29)	14(14/30)	15(15/35)		
III (*n* = 28)	13(13/30)	15(15/35)		
IV (*n* = 8)	3(3/30)	5(5/35)		
PAVW			−0.066	0.948
II (*n* = 25)	12(12/30)	14(14/35)		
III (*n* = 32)	15(15/30)	17(17/35)		
IV (*n* = 7)	3(3/30)	4(4/35)		
OLP			−0.085	0.932
II (*n* = 19)	10(10/30)	9(9/35)		
III (*n* = 3)	1(1/30)	2(2/35)		
IV (*n* = 0)	0	0		
SUI			−0.289	0.773
Light degree	6(6/30)	8(8/35)		
Moderate degree	3(3/30)	5(5/35)		
Occult SUI	7(7/30)	6(6/35)		

P*: Comparison between two group, *P* value obtained with Mann-Whitney *U* test or Wilcoxon *W* test.

### 3.3 Surgery-related evaluating indicators in different groups

The operation time, blood loss, post-operative hospitalized day, and operation complications all showed great significant difference between two groups (all *P*-values of <0.05) (Table 4). Although the patients of severe degree of SUI were not enrolled in this study, the incidence rate of SUI after operation showed a remarkable difference between two groups, with a rate of 26.67% (8/30) or 5.71%(2/35), respectively.

**Table 4:** Comparison of surgery-related evaluating indicators (*M* ± SD)

Variables	TVS group	NLS group	*t/*x* ^2^	*P*
Operation time (minute)	121.23 ± 13.88	91.09 ± 11.47	*9.45	0.000
Blood loss (milliliter)	107.53 ± 29.46	63.20 ± 12.85	*7.64	0.000
Postoperative hospitalized day (day)	7.30 ± 1.68	4.63 ± 0.84	*7.88	0.000
Operation complications (%)	12 (12/30)	5 (5/35)	5.53	0.019
Bladder injury	2	0		
Hematoma	4	2		
Urinary tract infection	4	1		
Voiding dysfunction	4	1		
Defecation dysfunction	3	2		
SUI	8(8/30)	2(2/35)	**5.448	0.035
Light degree	2	1		
Moderate degree	1	1		
Occult SUI	5	0		

*t: comparison by independent-samples *t*-test, *x* ^2^: Pearson’s *x* square test, **: Fisher’s exact test.

### 3.4 Scores of PFDI-20 between pre- and post-operation in different groups

The scores of post-operative overall PFDI-20 scores in 3 months and 6 months, respectively, showed great significance (all *P*-values = 0.000), and POPDI-6, CRADI-8, and UDI-6 all showed great difference between two groups (Table 5).

**Table 5:** Comparison of PFDI-20 of post-operation between different groups (*M* ± SD)

Follow-up time	3 months			6 months		
Variables	TVS group	NLS group	P*	TVS group	NLS group	P*
PFDI-20	44.50 ± 13.29	30.35 ± 16.86	0.008	30.48 ± 9.18	14.16 ± 10.58	0.000
POPDI-6	24.98 ± 6.65	17.37 ± 8.03	0.000	17.70 ± 5.79	8.86 ± 7.16	0.000
CRADI-8	4.11 ± 5.13	3.29 ± 5.14	0.000	2.20 ± 2.88	1.57 ± 2.65	0.000
UDI-6	14.58 ± 3.84	9.69 ± 8.97	0.000	10.03 ± 4.44	3.44 ± 4.15	0.000

P*: Comparison between two groups, *P* value obtained with *x* square test.

### 3.5 Pre- and post-operative POP-Q anatomic parameters

All anatomic parameters, including Aa, Ba, *C*, Ap, Bp, TVL, Gh, and Bp, showed great difference between pre- and 1 week post-operation in the same group (all *P* values = 0.000). All anatomic parameters in pre-operation showed no significant difference between two groups (all *P* values of >0.05), and except for parameter Gh, all parameters in post-operation also showed great difference between two groups (all *P* values of <0.001). But the anatomic parameter *D* also showed great significant difference between pre- and post-operation only in the NLS group, because of non-existent parameter *D* after hysterectomy. Furthermore, the TVL of post-operation between two groups showed great significant difference (*P* = 0.000). And in the NLS group, the length of post-operative TVL displayed 1.0–2.0 cm elongation compared with that in the TVS group (*P* value = 0.000) (Table 6).

**Table 6:** Comparison of different anatomic parameters of POP-Q^*^ between pre- and post-operation in different groups (M ± SD)

Variables	TVS group (*n* = 30)		NLS group (*n* = 35)		*P*-value of different analysis			
	Pre-	Post-	Pre-	Post	*P* ^a^	*P* ^b^	*P* ^c^	*P* ^d^
Aa (cm)	1.45 ± 1.04	−2.44 ± 0.44	1.63 ± 1.09	−2.84 ± 0.31	0.000	0.000	0.503	0.000
Ba (cm)	2.58 ± 0.94	−2.09 ± 0.58	2.37 ± 0.97	−2.7 ± 0.37	0.000	0.000	0.377	0.000
*C* (cm)	4.29 ± 1.18	6.37 ± 0.47	4.24 ± 1.48	−8.2 ± 0.96	0.000	0.000	0.896	0.000
TVL (cm)	7.08 ± 0.64	6.37 ± 0.47	7.21 ± 0.64	8.34 ± 0.85	0.000	0.000	0.417	0.000
Pb (cm)	3.00 ± 0.81	3.68 ± 0.53	3.11 ± 0.89	4.16 ± 0.34	0.000	0.000	0.593	0.000
Gh (cm)	4.33 ± 1.09	3.02 ± 0.38	4.20 ± 1.35	3.03 ± 0.21	0.000	0.000	0.667	0.874
Ap (cm)	1.47 ± 1.33	−2.49 ± 0.50	1.56 ± 1.22	−2.87 ± 0.28	0.000	0.000	0.775	0.000
Bp (cm)	2.33 ± 0.88	−2.03 ± 0.41	2.35 ± 1.05	−2.88 ± 0.26	0.000	0.000	0.910	0.000
*D* (cm)	3.17 ± 1.07	—	2.90 ± 0.94	−6.96 ± 0.70	—	0.000	0.275	—

^*^Pelvic organ prolapse quantification points, with measurements in cm relative to the position of the general hiatus.

Aa: a point located in the midline of the anterior vaginal wall, 3 cm proximal to the external urethral meatus.

Ba: the most distal/dependent point on the anterior vaginal wall from point Aa to the anterior vaginal fornix or vaginal cuff.

*C*: the most distal/dependent edge of the cervix or vaginal cuff (a measure of uterine descent).

*D*: the position of the posterior fornix.

Ap: a point located in the midline of the posterior vaginal wall, 3 cm proximal to the hymen.

Bp: the most distal/dependent point on the posterior vaginal wall above point Ap.

Pb: Perineal body. Gh: Genital hiatus. TVL: total vaginal length. cm = centimeter. –: no existed value.

^a^Comparison between pre- and post-operation in the TVS group, *P* value obtained with paired sample *T* test.

^b^Comparison between pre- and post-operation in the NLS group, *P* value obtained with paired sample *T* test.

^c^Comparison between two groups before operation, *P* value obtained with ANOVA.

^d^Comparison between two groups after operation, *P* value obtained with ANOVA.

### 3.6 Evaluation of the effect of LRAHUP by ultrasonography

**Figure 2:**
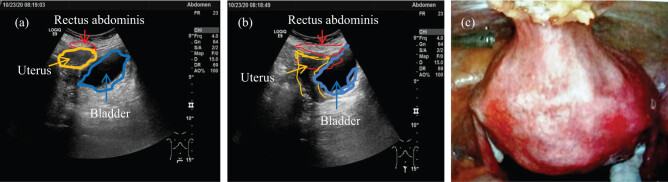
Ultrasound evaluation of postoperative effects of LRAHUP and clinical evidence of permanent adhesion.

Of 35 case patients who underwent LRAHUP, 91.43% (32/35) patients displayed good permanent adhesion between the anterior wall of the uterine body (yellow part) and the rectus abdominis (red part) at the end of 6-month follow-up after surgery (Figure 2a and b). And once this adhesion is formed, it is permanent and dense. This dense and permanent adhesion was also confirmed in laparoscopic surgery several years after cesarean section (Figure 2c).

### 3.7 Cumulative case number of postoperative recurrence cases of POP

**Figure 3:**
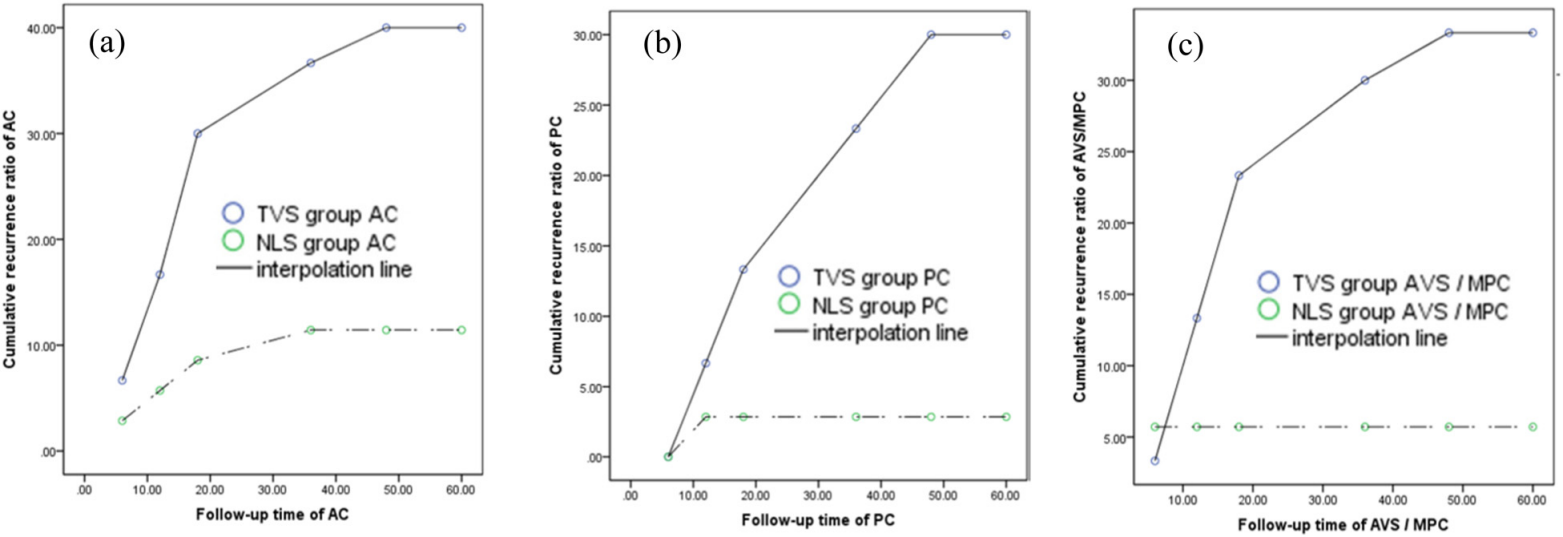
Relationship between the time of follow-up (lateral axis) and cumulative recurrence ratio (vertical axis).

In the TVS group, the cumulative recurrence ratio of AC, PC, and AVS/MPC after operation all showed a remarkable tendency, which showed first an increase and finally reached to a platform between 48- and 60-month follow-up period. But in the NLS group, AVS/MPC showed a steady platform cumulative recurrence ratio within a shorter 6-month follow-up period (Figure 3). Finally, cumulative numbers of post-operative recurrence in different variables AC, PC, and AVS/MPC all showed great difference between two groups (all *P*-values of <0.001) (Table 7).

**Table 7:** Cumulative recurrence ratio of different pelvic organ prolapsed in different follow-up time

Variables	6 months	12 months	18 months	36 months	48 months	60 months	*x* ^2^	P*
AC							7.106	0.008
TVS group	2/30	5/30	9/30	11/30	12/30	12/30		
NLS group	1/35	2/35	3/35	4/35	4/35	4/35		
PC							9.142	0.002
TVS group	0/30	2/30	4/30	7/30	9/30	9/30		
NLS group	0/35	1/35	1/35	1/35	1/35	1/35		
AVS/MPC							8.186	0.004
TVS group	1/30	4/30	7/30	9/30	10/30	10/30		
NLS group	2/35	2/35	2/35	2/35	2/35	2/35		

AC = anterior compartment, PC = posterior compartment, AVS = apex of vaginal stump, MPC = median pelvic cavity. P*: comparison of cumulative recurrence ration between two groups, *P* value obtained with *x* ^2^ test.

## 4 Discussion

Although the female POP is a common problem in gynecology, the surgically treated women for POP constitute only 11% [17]. In the past few decades, trans-vaginal hysterectomy was the standard surgical treatment for UP; however, more and more gynecologists found that vaginal hysterectomy could not solve the problems of UP and maybe elevate the recurrence rate of apex of vaginal stump or vaginal vault prolapse [1,2]. Recently, several operations have been proposed for the treatment of prolapsed uterus [18,19], and currently, between 31 and 60% of women in the United States presenting for prolapsed care would elect to keep their uterus if surgical outcomes were equally efficacious [20]. An 11-year large population-based descriptive study in Taiwan showed a trend of uterine suspension with uterine preservation during the latter years [9], and the uterus preservation is always considered when there is no pathological finding of uterus [4,11]. Our small series demonstrated that the technique of LRAHUP described earlier is theoretically feasible and safe in this study. First, rectus abdominis is a strong hard tissue, which is very suitable for the suspension of prolapsed uterus. Second, there were no important vessels and nerves around the anterior sheath of rectus abdominis; therefore, the “one-cut and two-tunnel” puncture method described earlier was safety and feasible. Third, this new procedure is completely in line with a contemporary famous theory proposed by DeLancey [21], which combined with not only repair of anterior or posterior vaginal wall but also with repair of laceration of perineum and Kelly’s operation (Kelly’s plication) when necessary (e.g., OLP, SUI). Finally, the dense adhesion of the anterior wall of the uterine body could be evaluated by a method called “sliding sign” [22], so we can infer that it has clear and reliable evidence of the expected results after suspension and fixation of uterus, that is to say, this suspension and fixation of uterus is temporary and the result of adhesion between the anterior wall of uterine body and the rectus abdominis is permanent (e.g., as shown in Figure 2a–c). The aforementioned theoretical analysis is enough to illustrate that LRAHUP can be feasible in theory and clinical practice in the future. And this result had also been proven by Long et al. [23] in China.

In this study, LRAHUP, as a new strategy for UP, was introduced in detail by comparison with traditional vaginal hysterectomy. It had shown many advantages of peri-operative outcomes, which were, respectively, shorter operation time, less blood loss, no longer post-operative hospitalization day, and lower post-operative complications. Especially for surgery-related complications, LRAHUP showed a simple, rapid, and safety surgical procedure with uterus-sparing and without the little chance of bladder injury (0%). Although the pre-experimental sample size is small and may affect the reliability of the finally obtained experimental data, all of these data were consistent with recently reported findings [23]. Moreover, LRAHUP also demonstrated good subjective satisfaction confirmed by the comparison of PFDI-20 scores between different groups in pre- and post-operation within 3- or 6-month follow-up. But the Female Sexual Function Index (FSFI) questionnaire of 65 patients before and after surgery was not included in this study; it may be the main reason that the enrolled patients had few or no desire for sex (e.g., husband death, single), because of an elder mean age of 65.83 and 64.71 years, respectively, for different groups. In addition, educational level, weak sexual awareness, and traditional sexual beliefs may also be some of the reasons not included in this study. It is recommended to include it in subsequent studies. Most interesting result is the lower incidence rate of SUI after operation in the NLS group, compared with that in the TVS group, although the same Kelly’s placation for SUI was performed on patients. This good result of lower incidence rate of SUI may be caused by the new technique of LRAHUP, which may help in indirect correction of SUI by the rectification of the mid-pelvic defects; while indirectly reinforcing the anterior pelvic cavity, the recurrence rate of SUI is indirectly reduced [24]. However, the exact reason for the lower incidence of SUI in the NLS group needs further study in terms of urodynamics.

The objective anatomic parameters also confirmed higher effective outcomes of an anatomical cure, including Aa, Ba, *C*, Ap and Bp, by comparison between pre- and post-operation in the same group, or between two groups in post-operation. And in the NLS group, the anatomic parameters all showed better advantages compared with those in the TVS group. Furthermore, concerning the anatomic parameter of TVL, the former showed a 1.0–2.0 cm elongation of TVL compared with that in the TVS group and this result was also confirmed by a recent study [23]. However, this good result of elongation of TVL improved the satisfaction of sex; unfortunately, this study did not involve. However, many scholars [25,26] had found that the impact of different methods of hysterectomy on vaginal length is in the following order: transvaginal > transabdominal > laparoscopic > robotic, while there was no significant difference in the impact on sexual activity between the two groups. In addition, post-operative dyspareunia is more common after vaginal hysterectomy compared to that after abdominal hysterectomy, This may be attributed to post-operative shortening of the vagina secondary to excessive trimming of the vaginal walls especially if VH was performed for utero-vaginal prolapse [27,28]. In summary, further research is needed to confirm the impact of extending vaginal length on patients’ sexual satisfaction, especially for young women.

Theoretically, uterine ventral suspension causes an upward traction of anterior vaginal wall, helping to correct the anterior compartment prolapse as well. However, there were many research reports that this suspension only created an anatomical cure rate of 85% in the anterior compartment [10], slightly lower than a recent study showing 91% following laparoscopic sacral hysteropexy [29]. This may be due to the fact that sole suspension of prolapsed uterus could not completely solve the problem of anterior compartment prolapse. Especially for those patients with anterior vaginal wall prolapse stage III (Ba > +1) pre-operatively, they showed a higher rate of recurrence [23]. A review study concluded that laparoscopic uterine ventro-suspension using round ligaments has a very limited role, with a success rate less than 50% [10]. It is apparent that elongation of round ligament by uterine weight causes a higher recurrence of UP. However, in this study, we performed on 65 patients with anterior vaginal wall prolapse stage II–IV not only uterus-sparing surgery of LRAHUP or vaginal hysterectomy but also anterior/posterior vaginal wall repair. Finally, results displayed that the different cumulative recurrence rates of AC/PC/MPC prolapse in the NLS group were at all remarkable lower levels compared with the TVS group after post-operation 5-year follow-up. Especially for the recurrence rate of AC/PC, 65 case patients who underwent the same procedure for AC/PC prolapse showed different results of clinical outcome. The above-described conclusions did not seem to accord with the clinical practice, because of which were the same procedure for anterior/posterior vaginal wall repair performed on 65 patients, and the sole different key point was the different procedure for prolapsed uterus between two groups. In other words, different procedures for UP with or without uterus-preserving POP surgery completely elaborated the reason for this above-described accordance with the clinical practice. It is well known that the correction of prolapsed MPC/AVS is a key and crucial problem; a good effective procedure for UP can help not only reduce the recurrence ratio of MPC but also lower the recurrence rate of AC/PC prolapse [30,31]. This evidence also reinforces the importance of safe and effective apical support and the clinical value of McCall or modified McCall procedures in preventing the occurrence of POP after hysterectomy because the principle of the McCall culdoplasty is to elevate the vaginal vault and obliterate the posterior cul-de-sac [29]. However, the preventive effect of McCall or modified McCall procedures on recurrence after hysterectomy in patients with POP remains to be further studied [32,33]. In this study, two strategies, namely, LRAHUP and traditional TVL for advanced UP, were all implemented. In addition, TVL procedure is relatively complex and difficult to perform in grassroots hospitals, and LRAHUP procedure is simple and easy to perform. Finally, the most important findings in this study showed that a longer follow-up period of 48–60 months in the TVS group according to the follow-up time taken to reach the platform, but only 6–12-month follow-up in the NLS group. This can guide and shorten the post-operative follow-up of time for LRAHUP.

In summary, the new technique of LRAHUP for uterus-sparing POP surgery, described and studied in detail, was confirmed a safety, simple, and effective procedure for those patients diagnosed with advanced UP, because of its safety, simplicity, feasibility, lower post-operative complications, better subjective assessment of PFDI-20 scores, better anatomic outcomes, elongation of TVL, lower cumulative recurrence ratio, and shorter follow-up period after operation. Although this study showed many good results, a little of flaw was displayed, including the absence of young pre-menopausal women patients and the FSFI questionnaire in this study. In particular, the overall improvement of quality of life and sexual function requires multidisciplinary management of POP, which has also attracted the attention of many clinical urologists and gynecologists [34]. So the disadvantages of the procedure of LRAHUP for uterus-preserving POP surgery still needed to be further studied with large sample and multicenters.

## 5 Conclusions

The LRAHUP may be a good procedure, because of its safety, simplicity, better anatomic outcome, better subjective assessment of PFDI-20 scores, elongation of TVL, lower cumulative recurrence ratio, and shorter follow-up period.
